# The Role of Integrin α_4_β_7_ in HIV Pathogenesis and Treatment

**DOI:** 10.1007/s11904-018-0382-3

**Published:** 2018-02-24

**Authors:** James Arthos, Claudia Cicala, Fatima Nawaz, Siddappa N. Byrareddy, Francois Villinger, Philip J. Santangelo, Aftab A. Ansari, Anthony S. Fauci

**Affiliations:** 1 Laboratory of Immunoregulation, National Institutes of Allergy & Infectious Diseases, National Institutes of Health, 10 Center Drive Rm 6A08, Bethesda, MD 20814 USA; 2 Department of Pharmacology and Experimental Neuroscience, University of Nebraska Medical Center, Omaha, NE 68198 USA; 3 New Iberia Research Center, University of Louisiana Lafayette, Lafayette, LA 70560 USA; 4 Walter H. Coulter Department of Biomedical Engineering, Georgia Institute of Technology, Atlanta, GA 30680 USA; 5 Department of Pathology & Laboratory Medicine, Emory University School of Medicine, Atlanta, GA 30322 USA

**Keywords:** HIV/SIV, GALT, Integrin α_4_β_7_, Inflammatory bowel disease, Mucosal transmission, Antiretroviral therapy

## Abstract

**Purpose of Review:**

Acute HIV infection is characterized by high-level viral replication throughout the body’s lymphoid system, particularly in gut-associated lymphoid tissues resulting in damage to structural components of gut tissue. This damage is irreversible and believed to contribute to the development of immune deficiencies. Antiretroviral therapy (ART) does not restore gut structure and function. Studies in macaques point to an alternative treatment strategy that may ameliorate gut damage. Integrin α_4_β_7_ mediates the homing of lymphocytes to gut tissues. Vedolizumab, a monoclonal antibody (mAb) antagonist of α_4_β_7_, has demonstrated efficacy and has been approved for the treatment of inflammatory bowel disease in humans. Here, we describe our current knowledge, and the gaps in our understanding, of the role of α_4_β_7_ in HIV pathogenesis and treatment.

**Recent Findings:**

When administered to macaques prior to infection, a nonhuman primate analogue of vedolizumab prevents transmission of SIV. In combination with ART, this mAb facilitates durable virologic control following treatment interruption.

**Summary:**

Targeting α_4_β_7_ represents a novel therapeutic approach to prevent and treat HIV infection.

## Introduction

A defining feature of acute HIV infection is high-level viral replication in gut-associated lymphoid tissue (GALT). The propensity of HIV to replicate in GALT was first recognized over 25 years ago [1]. The association of GALT with acute infection came from two independent studies carried out in an SIV/Rhesus macaque model [2, 3]. From these studies, it was noted that high-level viral replication in GALT is accompanied by a profound depletion of gut CD4^+^ T cells. Subsequently, it was demonstrated that HIV infection in humans leads to a similar loss of gut CD4^+^ T cells in the very early stages of infection [4–6]. This gut-tropic aspect of acute HIV infection is believed to play a central role in the development of immune deficiencies that define HIV disease. The rapid loss of CD4^+^ T cells is accompanied by damage to the structural integrity of the gut, which has been linked to chronic systemic immune activation [7, 8]. Thus, there is considerable evidence to suggest that events that occur in gut tissues in the first weeks of infection play a central role in AIDS pathogenesis [9] The development of effective antiretroviral therapies (ART) have proven to be extraordinarily effective in suppressing viral replication in HIV-infected individuals. ART delays the onset of HIV-mediated immune deficiencies and significantly extends the life of individuals infected with HIV. However, ART is associated with varying degrees of toxicity, and once it is withdrawn plasma viremia typically rebounds [10–12]. Furthermore, ART does not fully reverse the early damage to the structural integrity of the gut, nor does it allow CD4^+^ T cells in GALT to fully recover [13]. Chronic immune activation persists in patients, even in individuals in whom ART is administered shortly after infection [14]. In one recent study, initiation of ART as early as ~ 2 weeks postinfection did not prevent long-term and apparently irreversible damage to the gut [15].

In addition to replicating in GALT, HIV replicates in the peripheral lymph nodes, spleen, and other tissues and organs, and this replication also contributes significantly to HIV pathogenesis. Yet, it is generally recognized that the early infection and irreversible destruction of CD4^+^ T cells in GALT is a key event in the eventual development of immune deficiencies. Understanding the specific events surrounding this damage to gut lymphoid tissues may point to new and improved ways to prevent and treat HIV infection.

Migration of immune cells into and out of GALT is tightly regulated by receptors that control cell trafficking. Prominent among gut homing receptors is integrin α_4_β_7_ (α_4_β_7_). This heterodimeric receptor is comprised of a 180-kDa α chain (α_4_) and a 130-kDa β chain (β_7_). α_4_β_7_ is expressed on subsets of CD4^+^ and CD8^+^ T cells, B cells, NK cells, and macrophages. Both α_4_ and β_7_ can pair with other integrin chains; however, the α_4_β_7_ heterodimer is distinct in promoting trafficking of lymphocytes to GALT. The mechanism by which α_4_β_7_-expressing cells home to GALT involves a specific interaction with the mucosal addressin cell adhesion molecule (MAdCAM). MAdCAM in healthy adults is expressed on the cell surface of high endothelial venules (HEVs) of gut inductive sites and in the gut lamina propria. It is also found on the surface of follicular dendritic cells (FDCs) in mesenteric lymph nodes [16]. A majority of naïve, and a subset of memory, CD4^+^ T cells express α_4_β_7_, and these cells circulate throughout the peripheral lymphoid system. Importantly, it is the tissue-specific expression of MAdCAM that defines α_4_β_7_ as a gut homing receptor.

Integrins following ligation, including α_4_β_7_, are capable of delivering intracellular signals (outside-in signaling). Signaling is precisely coordinated with other integrins (α_4_β_1_ and LFA-1) and chemokine receptors in a multistep adhesion cascade that results in extravasation of α4β7 positive cells through venules and into gut tissues [17, 18]. However, α_4_β_7_ signaling is not limited to processes involving cell trafficking. Similar to α_4_β_1_ and LFA-1, α_4_β_7_ can provide costimulatory signals to CD4^+^ T cells [19–21]. Little is known regarding the context in which α_4_β_7_-mediated costimulation contributes to CD4^+^ T cell activation in vivo, although it is presumably most relevant in GALT [22].

## α_4_β_7_ and the Gut-Tropic Phenotype of HIV

A growing number of viruses have been shown to interact directly with integrins [23]. In some cases, the adaptive value of this interaction involves signal transduction that facilitates infection [23]. Certain rotaviruses as well as both HIV and SIV have been shown to engage α_4_β_7_ [21, 24–26]. For HIV and SIV, this interaction is mediated by the gp120 subunit of the envelope glycoprotein [24, 27, 28]. The identification of an interaction between HIV and α_4_β_7_ provided a new perspective to the earlier descriptions of the gut-tropic nature of HIV and SIV and the HIV/SIV-mediated pathologies that are associated with acute infection [29]. However, α_4_β_7_ is not required for entry into CD4^+^ T cells and its specific role in HIV pathogenesis remains under investigation.

## HIV and SIV gp120 Bind to and Signal Through α_4_β_7_

The gp120 subunit of HIV and SIV envelope proteins have been described as a series of five constant domains interspersed with five variable domains (C1, V1, V2, C2, V3, C3, V4, C4, V5, C5). The V2 domain of gp120 (V2-loop) is a ~ 50–90 amino acid region bounded by cysteines. It is contiguous with the V1 domain. The principal α_4_β_7_ binding site lies within V2 [21, 28, 30–32] and involves a conserved tripeptide motif, LDV/I. This tripeptide appears to function in a similar manner as tripeptide motifs encoded in MAdCAM (LDT), VCAM (IDS), and fibronectin (LDV), the three natural ligands of α_4_β_7_ [33]. All share a core aspartic acid that coordinates a Mg^++^ ion that sits in the metal ion dependent adhesion site (MIDAS) of the α_4_ chain. Metal ion coordination is required for binding of these natural ligands to α_4_β_7_, as well as for the binding of HIV and SIV gp120.

Binding of a recombinant HIV gp120 protein to α_4_β_7_ on primary CD4^+^ T cells rapidly activates LFA-1 [21]. LFA-1 activation, and possibly other aspects of gp120-mediated signal transduction through α_4_β_7_, may facilitate the formation of virological synapses. The utilization of an Asp-containing tripeptide, along with the requirement of a metal ion in mediating gp120 binding to α_4_β_7_, represents a striking example of molecular mimicry and suggests that HIV-α_4_β_7_ interactions evolved to serve an important role in HIV pathogenesis.

## α_4_β_7_^high^ Memory CD4^+^ T Cells Are Early Targets of HIV Infection

HIV gp120 V2 encodes a variable number of potential N-linked glycosylation sites (PNGs). Viruses isolated in the first months after infection can be distinguished from chronic isolates by tending to have fewer PNGs [34]. The removal of V2 PNGs enhances gp120 binding to α_4_β_7_ [27, 28]. Based on this observation, we proposed that α_4_β_7_^high^ memory CD4^+^ T cells are early targets of infection following mucosal transmission [35]. Subsequently, studies in the SIV/macaque model showed that α_4_β_7_^high^ memory CD4^+^ T cells are rapidly depleted following mucosal transmission of virus. Among the cells bearing this phenotype are TH-17 cells, which have independently been implicated as early targets of infection. Recently, Sivro and colleagues reported that, in HIV-infected women, α_4_β_7_^high^ memory CD4^+^ T cells are preferentially depleted from gut tissues as early as the first 2 weeks following infection [15]. That these cells might be among the first cells productively infected is further supported by the observation that they can be found in cervical cytobrush samples [36–38], and in both humans and macaques the frequency of α_4_β_7_^high^ memory CD4^+^ T cells in blood and cervical tissues increases in the context of sexually transmitted diseases and is associated with increased susceptibility to acquisition [39, 40]. In addition, the frequency of α_4_β_7_^high^ memory CD4^+^ T cells in mucosal tissues correlates with their frequency in blood. Blood frequencies range from ~ 7 to 25% and appear to be stable in healthy individuals over time. Interestingly, one study comparing matched groups of young, healthy African American and Caucasian men found that the frequencies of α_4_β_7_^high^ memory CD4^+^ T cells were higher in the African Americans [41], and suggested that this difference might contribute at least in part to the relatively increased incidence of HIV in this group of Americans. Thus, in vitro and in vivo studies point to a role for α_4_β_7_ in the initial stages of infection, which set the stage for studies aimed at determining whether targeting α_4_β_7_ could reduce the efficiency of viral transmission (see below).

## Development of an Anti-α_4_β_7_ Antagonist for Use in an SIV/Rhesus Macaque Model

Inflammatory bowel disease (IBD), which includes both Crohn’s disease and ulcerative colitis, afflicts over 1.3 million people in the USA. IBD is an autoimmune disease of unknown etiology localized primarily to the gut. One therapeutic strategy designed to suppress the inflammatory response associated with IBD involves administration of a humanized anti-α_4_β_7_ monoclonal antibody (mAb) [42–44]. This mAb, termed vedolizumab (Entyvio), is a derivative of a mouse mAb named Act-1 that was discovered more than 30 years ago [45, 46]. It was believed generally that Act-1 binds to an epitope shared between α_4_ and β_7_. However, based on crystallographic data [47], it has been proposed that Act-1 binds exclusively to the β_7_ chain of α_4_β_7_ in a way that specifically inhibits binding to MAdCAM.

Vedolizumab has proved to be effective in the treatment of IBD [42–44]. By blocking α_4_β_7_-MAdCAM interactions, it is thought that vedolizumab reduces trafficking of α_4_β_7_^high^ memory CD4^+^ T cells into GALT, which in turn should reduce localized inflammatory responses. Data exist to support this mechanism of action; however, the effects of vedolizumab are likely to be pleiotropic and other mechanisms have not been ruled out [48, 49]. Because of the tissue-specific expression of MAdCAM, vedolizumab does not exhibit the more potent and diffuse immunosuppressive effects associated with natulizumab, an α_4_ antagonist that targets both α_4_β_7_ and α_4_β_1_.

Act-1 does not block HIV infection of primary CD4^+^ T cells. In vitro, it has been shown to mediate modest reductions in the replication of some isolates; however, it has no impact on others [21, 31, 50]. The underlying mechanism of these effects is not known. Act-1 does, however, block binding of gp120 to α_4_β_7_, which we believe may have an impact on HIV pathogenesis. Considering the preferential infection of α_4_β_7_^high^ memory CD4^+^ T cells in the early stages of SIV/HIV infection, we reasoned that Act-1 might interfere with mucosal transmission. To test this hypothesis, we turned to an α_4_β_7_/SIV macaque model developed by Ansari and colleagues [51, 52].

In order to gain further insight into the role of GALT in HIV pathogenesis, Ansari and colleagues developed a primatized version of Act-1 [52]. The rationale underlying the development of this reagent was, in part, to determine whether reducing inflammatory responses in GALT would minimize HIV-mediated damage to gut tissues. This rationale is similar to the rationale for the use of vedolizumab in the treatment of IBD [53].

The primatized analogue of Act-1 has a serum half-life of 11.4 days. When administered intravenously at a dose of 50 mg/kg, it fully masked the expression of α_4_β_7_ expressed on the surface of lymphocytes harvested from colon biopsies [24, 51, 52]. Although the framework onto which the Act-1 complementary-determining regions (CDRs) were grafted was derived from a rhesus macaque IgG1 mAb, administration of the primatized anti-α_4_β_7_ (anti-α_4_β_7_ mAb) induces rhesus anti-rhesus antibodies (RARA), but at a low frequency (~ 20%). Our findings strongly suggest that RARA impacted treatment in an adverse way [54]. The frequency of anti-drug antibodies (ADA) in IBD patients treated with vedolizumab is low [53, 55–57].

## Treatment of SIV Infected Macaques with a Primatized Anti-α_4_β_7_ mAb

The initial evaluation of anti-α_4_β_7_ mAb in SIV infected macaques involved pre-treatment of macaques with the mAb 3 days prior and 3 weeks following IV inoculation with a high dose of SIVmac239. Animals received a second dose of mAb 3 weeks post-virus inoculation. No other drugs were included in this study. The viral inoculum was prepared as a stock using day 3 PHA activated rhesus PBMCs [51]. In untreated control macaques, infection with this virus typically leads to AIDS within 18 months. Relative to control animals, macaques receiving anti-α_4_β_7_ mAb exhibited significantly delayed peak SIV RNA and ~ 8-fold lower plasma set-point SIV RNA levels [51]. Of note, peak levels of viral RNA copies in jejeunal and colorectal biopsies were reduced by ~ 2 logs in the mAb-treated animals, and copies of proviral DNA in colorectal biopsies were low to undetectable. Thus, anti-α_4_β_7_ mAb was effective in reducing the amount of virus in GALT. Treatment also led to the preservation of peripheral CD4^+^ CCR5^+^ T cells. The impact of anti-α_4_β_7_ mAb on disease course was significant. While control animals developed AIDS 60–80 weeks after infection, treated animals remained healthy for 5 years (Ansari, unpublished).

In a recent study [58], we evaluated the frequencies of CD4^+^ T cells in colorectal biopsies from these anti-α_4_β_7_ mAb-treated animals. After an initial loss in the acute phase of infection, CD4^+^ T cells gradually increased over time, such that 60+ months postinfection, levels were close to that observed in healthy uninfected controls. This is notable in two ways. First, the effects of anti-α_4_β_7_ mAb were durable. Two infusions during acute infection were sufficient to dampen damage to gut tissues and preserve CD4^+^ T cells over an extended period of time. This preservation persisted despite the presence of significant plasma viremia, suggesting that a key aspect of anti-α_4_β_7_ mAb therapy may involve protection and/or restoration of the gut ultrastructure. Second, the capacity of a brief period of anti-α_4_β_7_ mAb therapy to alter the course of disease indicates that, at least in this SIV model, early virus replication in GALT and the consequent inflammation of gut tissues plays an important role in the development of disease many months later. It is important to keep in mind that the immune cell composition is different in small and large intestines [17, 59, 60], and are associated with different infections and pathologies [59]. We found that anti-α_4_β_7_ mAb treatment reduced virus in the colon to a greater extent than the small intestine [58].

## Anti-α_4_β_7_ mAb in the Prevention of Mucosal Transmission

McKinnon and colleagues isolated α_4_β_7_^high^ memory CD4^+^ T cells from cervical cytobrush specimens and found that they co-express multiple HIV susceptibility markers [37]. Moreover, α_4_β_7_ expression was correlated with CCR5 expression. These features would seem to make these cells favorable targets for productive infection. It is reasonable then to suggest that virus infects α_4_β_7_^high^ memory CD4^+^ T cells in the genital or rectal mucosa, and that these infected cells eventually migrate into GALT. An alternative way that virus might access gut tissues can be inferred from the recent description of the incorporation of α_4_β_7_ on the surface of free virus, and the capacity of virion-associated α_4_β_7_ to mediate virus adhesion to MAdCAM on HEVs [61]. For this to occur requires that virus first infects α_4_β_7_-expressing cells, which is consistent with the scenario outlined above.

With this scenario in mind, we asked whether anti-α_4_β_7_ mAb could reduce the efficiency of mucosal transmission of SIV. To address this question, we employed a stringent low-dose vaginal transmission model that utilized a highly infectious stock of SIVmac251. Animals were pretreated with anti-α_4_β_7_ mAb (50 mg/kg) or a control IgG mAb and then challenged weekly until 10 of 12 control animals were determined to be viremic. After 6 challenges, 6/12 anti-α_4_β_7_ mAb-treated animals remained uninfected while 10 of the 12 controls became infected. For those anti-α_4_β_7_ mAb-treated animals that did become infected, their infection was somewhat delayed since more total challenges were required than for animals in the control arm of the study [24].

The specific mechanisms by which animals were protected from infection remain unclear. Of note, anti-α_4_β_7_ mAb treatment did not alter the number of CD4^+^ T cells in the cervicovaginal compartment. However, it did mask > 99% of the α_4_β_7_ heterodimers present on the surface of cervicovaginal CD4^+^ T cells. One of the more intriguing results from this study was our finding that, in anti-α_4_β_7_ mAb-treated animals, but not in placebo controls, we could detect proviral DNA in cervical tissues 10 weeks after the last challenge. This would seem to indicate that anti-α_4_β_7_ mAb interfered with the egress of cells out of the vaginal tissue. MAdCAM is normally absent from the female genital tract. However, chlamydia infection of the female genital tract induces MAdCAM neo-expression [62–64], and one can speculate that SIV might promote a similar response. We proposed three mechanisms which alone or together may have reduced the efficiency of infection. Anti-α_4_β_7_ mAb may have interfered with: (1) trafficking, by blocking α_4_β_7_ binding to MAdCAM; (2) V2 peptide interacting with α_4_β_7_; and (3) signaling through α_4_β_7_ on CD4^+^ T cells, either by MAdCAM or V2.

There exist significant gaps in our understanding of the early events that lead to irreversible SIV/HIV infection. Our demonstration that anti-α_4_β_7_ mAb can prevent infection, at least in the context of vaginal transmission, argues that α_4_β_7_-expressing cells play an early role in establishing infection. Key questions remain, including whether α_4_β_7_ is involved in rectal transmission. Martinelli and colleagues report that the susceptibility of macaques to rectal SIV challenge correlates directly with the frequency of α_4_β_7_^high^ memory CD4^+^ T cells in rectal tissue [65], suggesting that this might be the case. Finally, it remains to be determined whether α_4_β_7_-expressing cells contribute to long-lived viral reservoirs, and if treatment with α_4_β_7_ antagonists can alter the size and/or durability of those reservoirs.

## α_4_β_7_ Antagonists as Adjunctive Therapies to ART

Current ART therapies are highly effective in allowing subjects to control viremia for an extended period of time. Yet, the persistence of a viral reservoir, despite ART, requires that the vast majority of HIV-infected individuals remain on therapy indefinitely. Moreover, ART does not fully resolve the chronic immune activation associated with infection. Given the demonstrated capacity of anti-α_4_β_7_ mAb to promote durable control of viremia in SIV-infected macaques in the absence of ART [24, 51], it is reasonable to ask whether α_4_β_7_ antagonists might prove useful as an adjunctive therapy to ART. Unlike many other proposed adjunct therapies, the clinical development of α_4_β_7_ antagonists is well underway. The demonstrated efficacy of vedolizumab in the treatment of IBD has catalyzed efforts aimed toward the development of new and hopefully more effective α_4_β_7_ antagonists [42].

To explore how an α_4_β_7_ antagonist might compliment ART, we employed a stock of SIVmac239 prepared as described by Derosiers et al. [66]. Considering the cumulative impact of infection on the immune system over time, we designed a protocol in which infection was established, but the immune system remained partially competent. Animals were placed on ART 5 weeks postinfection around the time that set-point viral load is typically established [54]. Four weeks later, the first of eight anti-α_4_β_7_ mAb infusions and, for purposes of control, normal IgG infusions, were administered to two groups of Rhesus macaques. Nine weeks later, ART was terminated. While 7/7 of the animals that received ART and a control IgG mAb showed significant plasma viral rebound, 8/8 of the animals that received ART + anti-α_4_β_7_ mAb controlled plasma viremia to either low or undetectable levels. Control of viremia in the anti-α_4_β_7_ mAb arm persisted in all animals for > 3 years, with some animals showing intermittent blips in plasma viremia. CD4^+^ T cells counts in blood were restored to near pre-infection levels, and gut CD4^+^ T cells also recovered. In brief, the addition of an anti-α_4_β_7_ mAb to a standard form of ART promoted durable control that has allowed these animals to control viremia for an extended period following withdrawal of all therapy. Although plasma aviremic, each of these animals remain infected, and studies are ongoing to identify the immune mechanisms responsible for viral control.

**Fig. 1:**
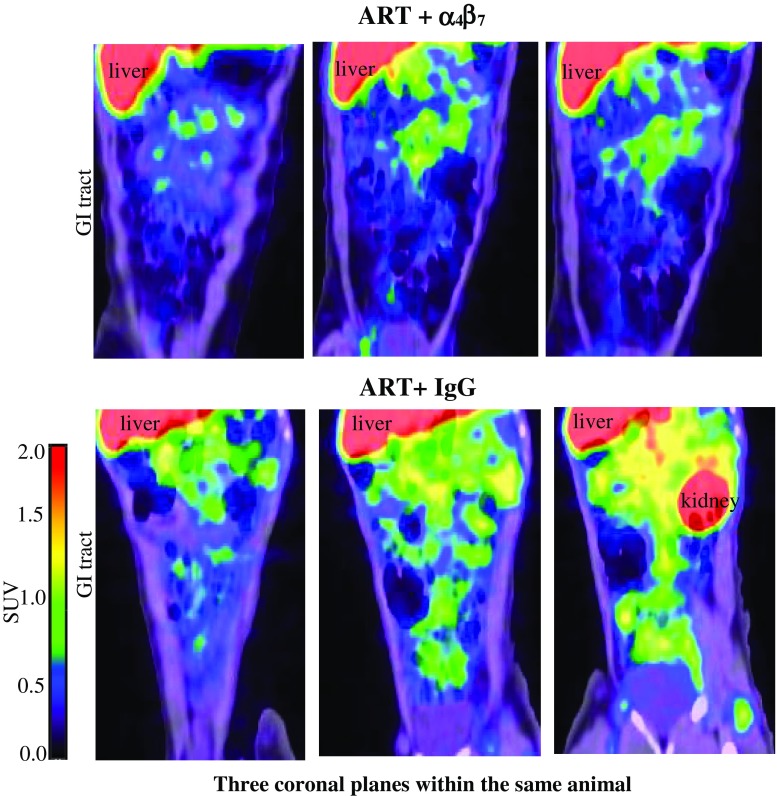
Immuno-PET/CT analysis of SIV gp120 in animals following treatment with ART+ anti α_4_β_7_ mAb. Comparison of gp120 signals in gut tissues of an ART + α_4_β_7_ mAb-treated animal (RLN12, upper) and an ART-only animal (RuS14, lower) at 34 and 39 weeks postinfection, respectively. Three coronal planes of the GI tract from each animal are shown. Immuno-PET images acquired as described in Santangelo et al. [58]. Further details in Byrareddy et al. [54]

In attempts to identify the mechanisms that mediated viral control, we focused on the dual therapy period when both ART + IgG and ART+ anti-α_4_β_7_ mAb-treated animals were plasma aviremic, reasoning that the immune responses that would eventually control viral replication in the animals that received anti-α_4_β_7_ mAb likely developed during this period. Among the most notable differences between the two groups during the dual therapy phase of the study was a relative increase in CD4^+^ T cells in the animals receiving anti-α_4_β_7_ mAb. These increases were observed in both blood and colorectal tissues, and included Th17/22 cells. Loss of Th17/22 cells is noteworthy because it is correlated with increased damage to GALT [67]. Plasma I-FABP levels, which also correlate directly with gut damage, began to decrease during the dual therapy phase of the study in the animals receiving anti-α_4_β_7_ mAb. In a follow-up study, that employed PET/CT image analysis, we determined that the addition of anti-α_4_β_7_ mAb reduced the number of infected cells in gut tissues relative to ART alone during the dual therapy period [58]. Given that ART alone efficiently suppresses plasma viremia, it is unclear how anti-α_4_β_7_ mAb was able to reduce the number of infected cells in gut tissues beyond that achieved by ART; however, this reduction is likely relevant to the durable control that we observed in these animals. Interestingly, following interruption of both ART and anti-α_4_β_7_ mAb the reduction of infected cells in gut tissues persisted (Fig. 1). Other differences that appeared during the dual therapy period included increases in colonic NKp44^+^ ILCs [54, 68, 69], and levels of plasma retinoic acid (ATRA) in the anti-α4β7 mAb-treated group. Interestingly, plasma TGF-β levels were significantly lower in the ART+ anti-α_4_β_7_ mAb-treated animals compared to the ART + control IgG group during the dual therapy phase of treatment.

Regarding adaptive immune responses, we could find no evidence of CD8^+^ T cell activity that distinguished the two treatment groups. None of the animals generated neutralizing antibody responses. However, all eight of the animals receiving anti-α_4_β_7_ mAb developed measurable antibody responses to a 15 amino acid region within the V2 domain, while only 3/7 ART+ IgG animals generated a similar response. Interestingly these V2 antibodies block SIV gp120 binding to α_4_β_7_ (submitted).

Elucidating the mechanisms of control in ART+ anti-α_4_β_7_ mAb-treated macaques remains a work in progress. It will be important to better understand which features of the experimental design were critical to achieving control and whether the protocol we employed can be further optimized. Is the control these animals achieved restricted to the stock of SIVmac239 or can it be extended to other SIV isolates? Would control be achieved if the animals were left untreated with anti-α4β7 mAb for a longer period of time? It is possible that if infection was allowed to go untreated into the chronic phase of infection that the immune cells providing control would be disabled. Conversely, how much viral antigenic experience is required for control? Would control be achieved if monkeys were placed on ART earlier than 5 weeks postinfection, when the viral antigenic load was lower? Does control require the anti-α_4_β_7_ mAb we employed, or would other α_4_β_7_ antagonists also promote durable control? Contrary to vedolizumab, whose Fc component was altered and disabled for use in humans, the Rhesus mAb we employed retains Fc effector functions. Were those functions relevant to control? Lastly, will similar results be obtained in humans treated with an α_4_β_7_ antagonist, whether they start ART in acute or chronic HIV infection? These and other questions await further investigation.

## Conclusion

HIV and SIV are frequently viewed as gut-tropic viruses, at least in the early stages of infection. This concept stems from observations made over 15 years ago that, soon after infection, high levels of viral replication occur in GALT and a large fraction of gut CD4^+^ T cells are consequently lost. It is now possible to refine this concept. Studies from human cohorts and nonhuman primates have shown, in a convincing way, that α_4_β_7_^high^ memory CD4^+^ T cells are highly susceptible to infection. It is therefore more accurate to view HIV and SIV as viruses that exhibit a tropism for this subset of CD4^+^ T cells, and as a consequence of this cellular tropism viral replication is rapidly established in GALT. Understanding HIV in this way provides a potential target for both prevention of infection, and treatment after infection. Nonhuman primate studies support α_4_β_7_ as a target for both. To fully understand the role of α_4_β_7_ in pathogenesis and take full advantage of it as a therapeutic target, it is important to consider several points. First, α_4_β_7_ is a structurally dynamic receptor that mediates outside-in signaling to cells. Second, the HIV envelope protein gp120 binds to and signals through α_4_β_7_. Finally, the development of α_4_β_7_ antagonists for the treatment of IBD is well underway, with one drug already approved. To determine if and how these antagonists can be used in the treatment of HIV infection, it is critical that we increase our basic understanding of how α_4_β_7_ contributes to HIV pathogenesis.
